# Electrical-gain-assisted circularly polarized photodetection based on chiral plasmonic metamaterials

**DOI:** 10.1038/s41377-025-01932-9

**Published:** 2025-08-11

**Authors:** Chenghao Chen, Zhenhai Yang, Tianyi Hang, Yining Hao, Yijing Chen, Chengzhuang Zhang, Jiong Yang, Xiaoyi Liu, Xiaofeng Li, Guoyang Cao

**Affiliations:** 1 School of Optoelectronic Science and Engineering & Collaborative Innovation Center of Suzhou Nano Science and Technology, Soochow University, Suzhou, China; 2 Key Laboratory of Advanced Optical Manufacturing Technologies of Jiangsu Province & Key Laboratory of Modern Optical Technologies of the Ministry of Education, Soochow University, Suzhou, China; 3 Engineering Research Center of Digital Graphic and Next-Generation Printing of Jiangsu Province, Soochow University, Suzhou, China

**Keywords:** Optics and photonics, Electronics, photonics and device physics

## Abstract

Circularly polarized light (CPL) detectors based on chiral organic materials or inorganic structures hold great potential for highly integrated on-chip applications; however, these devices usually have to seek an optimal balance among the asymmetry factor (*g*), responsivity (*R*), and stability. Here, we aim to break such a limitation by combining chiral inorganic plasmonic metamaterials with electrical gain, by which one can enhance both *g* and *R* while simultaneously securing the stability. We demonstrate a CPL detector based on “S”-shaped chiral Ag nanowires/InAs/Si heterostructures, where the meticulous construction of the “S”-shaped chiral Ag nanowires with the overlaying InAs channel enables a substantial absorption asymmetry in InAs due to differentiated localized surface plasmon resonances excited by left- and right-circularly polarized (LCP and RCP) light. The InAs serves as a conductive channel, achieving significant electrical gain through photoconductive effects assisted by photogating, gate modulation, and trap effects. The proposed inorganic stable device exhibits a high electrical *g* of ~1.56, an ultra-high *R* of ~33,900 A W^−1^, a large specific detectivity of ~1.8 × 10^11^ Jones, and an ultra-short response time of ~23 ns, with the high performance achieved in a broad spectral range from 2 μm to 2.8 μm. Ultimately, by encoding ASCII code 1 and 0 onto LCP and RCP light, respectively, and leveraging the device’s heightened discrimination and response performance to these polarizations, we demonstrate a simple yet key-free optical encryption communication scheme at the device level, highlighting its extensive potential for system-level applications.

## Abstract (web summary)

This work proposes an advanced strategy that combines chiral inorganic metamaterials with electrical gain mechanisms to achieve simultaneous enhancement of the responsivity and the asymmetry-factor in circularly polarized light detectors, while ensuring stability.

## Graphical abstract

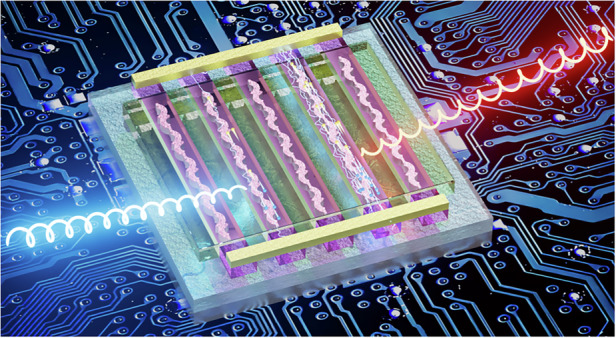

## Introduction

Chirality is a universal phenomenon in nature, manifesting across diverse scales, from microscopic molecules to vast cosmic nebulae^1,2^. The fundamental chiral states of electromagnetic fields, specifically left- and right-circularly polarized (LCP and RCP) light, exhibit strong interactions with chiral materials^3^. Such distinctive interactions have emerged as powerful tools for the detection and analysis of chiral molecules^4^. Circularly polarized light (CPL) detectors have been extensively employed in applications such as biosensing^5^, molecular detection^4^, and circular dichroism spectroscopy^6,7^, demonstrating exceptional versatility and precision. Furthermore, CPL detectors have found utility in advanced fields such as optical communication^8,9^, imaging technologies^10^, and quantum computation^11^, underscoring their broad potential across various disciplines. However, traditional CPL detectors rely on polarizers and quarter-wave plates^12^, which hinder device miniaturization and integration. In recent years, filterless CPL detectors based on chiral materials/structures have garnered significant attention due to their ability to directly detect CPL, thereby offering promising avenues for highly integrated on-chip systems^13–40^.

**Fig. 1:**
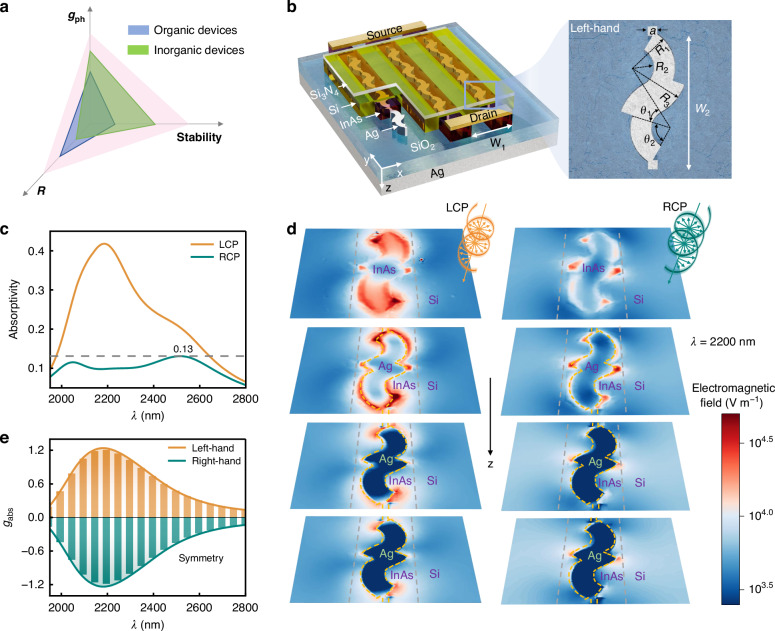
Structural diagram and optical performance of the “S”-shaped chiral Ag nanowire/InAs/Si-based CPL detector. **a** Comparison of *g*_ph_, *R*, and stability of current CPL detectors based on organic chiral materials and inorganic chiral structures. **b** Structural diagram of an “S”-shaped chiral Ag/InAs/Si-based CPL detector, with a detailed view of the chiral Ag nanostructure, where *θ*_1_ = 20° and *θ*_2_ = 100°, *R*_1_ = 130 nm, *R*_2_ = 60 nm, *R*_3_ = 170 nm, *a* = 30 nm, *W*_1_ = 560 nm, and *W*_2_ = 460 nm. **c** Absorptivity spectra of the InAs layer, and **d** spatial distributions of electromagnetic field intensity in the left-handed Ag nanowires-based CPL detector under LCP and RCP light incidence. **e** The *g*_abs_ spectra of CPL detectors based on left-handed and right-handed Ag nanowires

Existing CPL detectors based on chirality can be divided into two main types. The first type is based on chiral organic materials, which were used for direct detection of CPL due to the intrinsic chirality, including chiral organic small molecules^13–23^, chiral supermolecules^24^, and chiral organic-inorganic hybrid perovskite materials^25–35^. For instance, Campbell et al. reported a CPL detector based on the chiral small molecules 1-aza[6]helicene, demonstrating for the first time a significant difference in photocurrent response to RCP and LCP light. However, the device displayed low responsivity (*R*) of 0.01 A W^−1^ and failed within milliseconds^22^. Similarly, Tang et al. showcased a CPL detector based on chiral organic-inorganic hybrid perovskites, achieving a high *R* value of 0.797 A W^−1^ due to the excellent photoelectric properties of perovskite materials. However, it also exhibited a low *g*_ph_ of 0.1 because of the intrinsically weak chiral response, and a limited stability of just one month^30^. Note that the electrical asymmetry factor *g*_ph_ = 2(*I*_L_ − *I*_R_)/(*I*_L_ + *I*_R_), which ranges from −2 to 2 [where *I*_L_ (*I*_R_) is the response current of the device for LCP (RCP) light], quantifies the device’s ability to distinguish between LCP and RCP light. Despite their capability for direct CPL detection, organic chiral materials-based CPL detectors typically suffer from low *g*_ph_ due to the weak intrinsic chirality of these materials, and most notably, inherently poor stability, as illustrated in Fig. 1a, significantly constraining their practical applications.

To address these limitations, a second category of CPL detectors based on inorganic chiral plasmonic metamaterials has been developed^36–40^. These detectors utilize chiral metallic micro- and nanostructures to differentially absorb LCP and RCP light, thereby enhancing the response difference while maintaining stability. However, their *R* remains a significant challenge (see Fig. 1a), resulting from the inefficient injection of hot electrons generated in the metal into the semiconductor materials. For instance, Li et al. devised a CPL detector employing a “Z”-shaped chiral silver (Ag) nanoarray/silicon (Si) structure, achieving a notable *g*_ph_ of ~1.1 at 1350 nm, but with a low *R* of only 10^–3^ A W^−1^ ^36^. Similarly, Xiao et al. attempted to enhance *R* by embedding a chiral structure, but only achieved an enhancement to 0.021 A W^−1^ ^37^. In summary, the direct detection of CPL utilizing chiral materials/structures remains in its nascent stages, confronted with the intricate challenge of balancing *g*_ph_, *R*, and stability. Current strategies fall short of meeting the requirements for practical applications, necessitating the urgent need to develop CPL detectors that concurrently exhibit high *g*_ph_, *R*, and stability.

In this study, we propose a CPL detector that integrates “S”-shaped chiral Ag nanostructures with an inorganic indium arsenide (InAs)/Si heterojunction. The “S”-shaped chiral Ag nanostructures effectively excite localized surface plasmon resonance (LSPR), enabling differential modulation of the absorption of InAs for LCP and RCP light. Meanwhile, InAs serves as the channel, enabling significant electrical gain through mechanisms such as the photoconductivity effect, photogating effect, gate modulation, and trap effect. Through rigorous multiphysics coupling simulations, we demonstrate that the proposed inorganic device achieves simultaneously a high *g*_ph_ of 1.56 and an ultrahigh *R* of 33,900 A W^−1^, accompanied by a broadband response spanning 2000–2800 nm in the near-to-mid-infrared region. Furthermore, the InAs/Si heterojunction configuration also enables the device to operate in a self-powered mode, featuring an ultrafast response time of 10 ps and a high specific detectivity of 8 × 10^13^ Jones. Leveraging the outstanding performance of the proposed CPL detector, we further construct a simplified and keyless optical encryption communication system, showcasing the device’s versatile functionalities and practical potential. The proposed strategy of coupling chiral plasmonic metamaterials with electrical gain, offers a promising pathway towards realizing high-performance CPL detection.

## Results

### Structure and principle

The architecture of our proposed CPL detector is illustrated in Fig. 1b. The device is constructed on an Ag substrate, which is overlaid with a SiO_2_ dielectric layer. This bilayer configuration primarily functions as a rear reflector to enhance the optical absorption efficiency of the device. A periodic array of “S” like-shaped chiral Ag nanowires is positioned atop the SiO_2_ layer. A detailed view of these chiral Ag nanostructures is shown in Fig. 1b. This chiral nanostructure exhibits left-handed chirality, rendering it more sensitive to LCP light. Conversely, a right-handed chiral structure, as the mirror image of its left-handed counterpart, exhibits greater sensitivity to RCP light. The InAs nanowire shells serve as the photosensitive layer, covering the chiral Ag nanowires. Notably, the InAs nanowires are designed to extend beyond their ends to facilitate electrical connections with the source and drain electrodes. Afterward, a Si layer is introduced as a semiconductor dielectric to cover the InAs nanowires and fill the gaps in the nanowire array. To form a heterojunction for reducing the dark current of the InAs channel, the InAs and Si layers are lightly doped with *n*-type and *p*-type dopants with concentrations of 10^15^ and 10^16 ^cm^–3^, respectively. A Si_3_N_4_ film covers the Si layer, serving as an insulating protective coating.

The design principle for this work is as follows. A meticulous analysis of the formulations governing *R* and the *g*-factor reveals that there is no inherent trade-off between these two parameters. Specifically, the proportional enhancement of *I*_L_ and *I*_R_ can boost *R* without compromising the value of *g*_ph_. Consequently, incorporating electrical gain into inorganic chiral plasmonic metamaterials-based CPL detectors is a promising strategy for simultaneously achieving high *g*_ph_ and *R*. However, there are few reports on this approach to date, as its effectiveness strongly depends on the ingenious coupling of chiral metamaterial design, the overall device structure, photosensitive materials, response wavebands, and the electrical mechanisms, ensuring that the semiconductor channel exhibits electrical gain, and significant absorption asymmetry between LCP and RCP light. Here, our proposed “S”-shaped chiral metamaterial exhibits differentiated LSPRs excited by LCP and RCP light, and the overlay structure further enhances the absorption asymmetry in the InAs channel within the near-to-mid-infrared spectral range, thereby unlocking the potential for the device to simultaneously achieve high *g*_ph_ and *R* under electrical gain mechanisms. To validate the feasibility of this scheme, we develop a rigorous three-dimensional multiphysics-coupled simulation model based on the finite element method, enabling an accurate assessment of device performance. By rigorously solving Maxwell’s equations and semiconductor continuity equations in a coupled manner, the photoelectric response of the device is accurately modeled. Details of the simulation methodology are outlined in the Method section, with key material parameters used for this simulation listed in Table S1 (Supporting Information). In this simulation, a beam of LCP or RCP light is vertically illuminated onto the top surface of the device. The chiral Ag nanowires induce differential light absorption in the InAs layer for LCP and RCP light, leading to distinct photocurrent responses in the device under an applied source-drain voltage (*V*_ds_). To achieve significant absorption differences, the device structure needs to undergo optimization. Detailed optimization processes are provided in the Supporting Information (Section S1). The final optimized parameters are as follows. For the Ag nanowires, the thickness, and periods (*W*_1_ and *W*_2_) are 120 nm, 560 nm and 460 nm, respectively, with *θ*_1_ = 20° and *θ*_2_ = 100°, *R*_1_ = 130 nm, *R*_2_ = 60 nm, *R*_3_ = 170 nm, and *a* = 30 nm; the thickness and width of the InAs nanowires are 140 nm and 180 nm, respectively; the thickness of Si is 340 nm. It should be noted that the thicknesses of Ag, InAs, and Si are all measured from the top surface of the SiO_2_ layer as the reference plane. The thicknesses of the SiO_2_ and Si_3_N_4_ layers are 150 nm and 50 nm, respectively. Furthermore, the device exhibits excellent dimensional robustness, which will be discussed in subsequent sections.

### Optical asymmetry

For the left-handed Ag nanowire-based CPL detector, the absorptivity spectra of the InAs layer under LCP and RCP light incidence are shown in Fig. 1c. The absorptivity of the InAs for LCP light is significantly higher than that for RCP light within a broad near-to-mid infrared wavelength (*λ*) range of 1950 nm to 2800 nm. For LCP light, the maximum absorptivity exceeds 0.4 at a *λ* of about 2200 nm, while for RCP light, the absorptivity remains below 0.13 across the investigated waveband. To further elucidate this phenomenon, we analyze the distribution of the electromagnetic field intensity across four cross-sections of the device under illumination at *λ* = 2200 nm, as depicted in Fig. 1d. Specifically, the uppermost cross-section is located within the InAs layer, while the remaining three cross-sections are positioned at the top, middle, and bottom regions of the Ag nanowire. The results clearly indicate that under LCP light incidence, the electromagnetic field intensity within both the left-handed Ag nanowire and the InAs layer is markedly stronger than that under RCP light incidence. This is attributed to that the left-handed nanostructure exhibits higher sensitivity to LCP light, thereby exciting a more intense LSPR. In addition, as shown in Fig. S2, the absorption spectra of devices without chiral Ag nanowires demonstrate identical InAs absorptivity under LCP and RCP illumination, effectively ruling out the influence of InAs material anisotropy; the resonance peak shift in the absorption spectra further confirms the LSPR-mediated resonance modulation.

To quantify this optical asymmetry, we calculate the optical asymmetry factor *g*_abs_, defined as 2(*A*_L_InAs_ − *A*_R_InAs_)/(*A*_L_InAs_ + *A*_R_InAs_), where *A*_L_InAs_ (*A*_R_InAs_) is the absorptivity of the InAs layer under LCP (RCP) light incidence. Figure 1e displays the *g*_abs_ values for the CPL detectors based on left-handed Ag nanowires. Notably, the absolute values of *g*_abs_ exceed 0.2 across the *λ* range of 1950 nm to 2800 nm, yielding a peak value of 1.24 at *λ* = 2200 nm, which outperforms other reported CPL detectors^13,15,25,37^. Moreover, the *g*_abs_ of both left-handed and right-handed devices exhibit a symmetric distribution, validating the accuracy of the simulation results. The corresponding absorptivity spectra for the CPL detector based on right-handed Ag nanowires are provided in Fig. S2. Additionally, the dimensional robustness of the device is also assessed, as outlined in section S3 of the Supplementary Materials, demonstrating that *g*_abs_ remains stable across a wide range of InAs and Si geometric sizes, which facilitates practical device fabrication. The significant selective optical absorptance of the InAs layer in the proposed CPL detector ensures a notable selective electrical response.

### Electrical asymmetry and gain

**Fig. 2:**
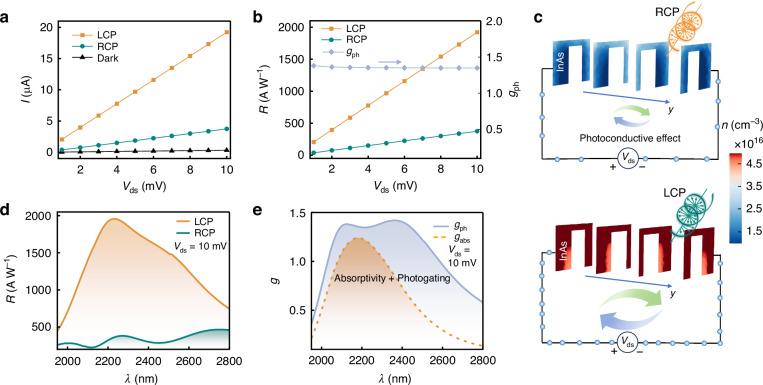
Electrical asymmetry of the proposed CPL detectors. **a** The *I*–*V* characteristics of the device under dark conditions and LCP/RCP light incidence, with a *λ* of 2200 nm and a light power density of 38,820 W m^−2^. **b** The *R* and *g*_ph_ of the device as a function of *V*_ds_. **c** Schematic of the photoconductive effect and the spatial distributions of electron concentrations (*n*) within the InAs layers under LCP and RCP light incidence with *λ* = 2200 nm. Here, four typical cases under different depths are considered. **d** The *R* of the device as a function of *λ* under LCP and RCP light incidence at *V*_ds_ = 10 mV. **e** The *g*_ph_-*λ* relationship of the device at *V*_ds_ = 10 mV, where the *g*_abs_ is also presented as a comparison

Based on the optical results and the fine symmetry of the left- and right-handed devices, we focus on the left-handed devices when investigating their electrical performance. Figure 2a illustrates the current–voltage (*I*–*V*) characteristics of the device under LCP and RCP light incidence at *λ* = 2200 nm, with the dark-state current as a reference. Notably, the linear increase in both illuminated and dark-state response currents with increasing *V*_ds_ indicates robust ohmic contacts between the InAs and the source/drain electrodes and confirms the efficient operation of the photoconductive effect. Furthermore, the distinct current responses to LCP and RCP light underscore the device’s capability to distinguish between these two polarization states. Figure 2b illustrates that the *R* under both LCP and RCP light increases with *V*_ds_, reaching 1921.6 and 374 A W^−1^, respectively, at *V*_ds_ = 10 mV, while the *g*_ph_ almost remains constant at ~1.35. The differentiated electrical response originates from the variation in photogenerated carrier concentrations in the InAs channel, as illustrated in Fig. 2c. Specifically, the electron concentration in the InAs channel under LCP light is significantly higher than that under RCP light, which is a consequence of the light absorption differences. The high *R* stems from the electrical gain induced by the photoconductive effect, which can be obtained from the theory^41^:

1 $$R=\frac{I-{I}_{\mathrm{dark}}}{{P}_{\mathrm{in}}S}=\frac{{qA}}{h{\rm{\nu }}}\cdot \frac{({\mu }_{{\rm{n}}}+{\mu }_{{\rm{p}}})\tau {V}_{\mathrm{ds}}}{{L}^{2}}$$

2 $${G}_{{\rm{a}}}=\frac{({\mu }_{{\rm{n}}}+{\mu }_{{\rm{p}}})\tau {V}_{\mathrm{ds}}}{{L}^{2}}=\tau \left(\frac{1}{{\tau }_{\mathrm{tn}}}+\frac{1}{{\tau }_{\mathrm{tp}}}\right)$$

where *I*_dark_, *P*_in_, *S*, *q*, *A*, *h*, *ν*, *μ*_n_ (*μ*_p_), *τ*, *L*, *τ*_tn_(*τ*_tp_) represent the dark current, incident optical power density, photosensitive area, electron charge, absorptivity, Planck’s constant, photon frequency, electron (hole) mobility, carrier lifetime, channel length, and electron (hole) transit time across the channel, respectively. *G*_a_ is the electrical gain, defined as the ratio of carrier lifetime to the transit time, highlighting that the ability of carriers to traverse the channel multiple times before recombination due to electrical neutrality principles. This behavior, depicted in Fig. 2c, leads to a high response current. This invariance of *g*_ph_ arises from the proportional amplification of both *I*_L_ and *I*_R_ as the bias voltage increases, maintaining a constant ratio of *I*_L_ and *I*_R_ (please refer to the expression of *g*_ph_). This observation confirms the conclusion mentioned above that there exists no inherent trade-off between *R* and *g*_ph_, emphasizing the potential of the device to achieve both high *R* and *g*_ph_ simultaneously.

We further delve into the spectral response of the device. Figure 2d illustrates the relationship between *R* and *λ* under LCP and RCP light incidence at *V*_ds_ = 10 mV. It is evident that, on the one hand, a pronounced difference in *R* between LCP and RCP light is observed across a broad range of near-to-mid infrared wavelengths. On the other hand, the *R* values remain relatively high for both LCP and RCP light. Specifically, under LCP incidence, the *R* exceeds 500 A W^−1^ almost consistently within the wavelength range of 1950–2800 nm, with a peak value reaching 1950 A W^−1^ at *λ* = 2230 nm. Conversely, under RCP incidence, the *R* is confined within the range of 224–466 A W^−1^. It is worth noting that the *R* can be further enhanced by increasing *V*_ds_, as demonstrated in Eq. (1). However, this voltage range for a linear relationship is ultimately limited by the saturation velocity. Specifically, at low *V*_ds_, the carrier drift velocity exhibits a linear relationship with the applied voltage, thereby preserving linear behavior. As voltage increases, the drift velocity approaches the thermal velocity (*v*_s_), causing the current–voltage dependence to deviate from linearity. Through calculation, we derive an approximate voltage range of 0–88.6 mV for the linear operation region. (Please see the section S4 for specific calculations) Fig. 2e showcases the *g*_ph_ within the same waveband, indicating that the *g*_ph_ is almost consistently exceeding 0.4, peaking at 1.4 at *λ* = 2400 nm, which signifies superior performance compared with the current CPL detectors^13,15,27,36^. Moreover, the device achieves a bandwidth spanning 500 nm (from 2030 nm to 2590 nm) for *g*_ph_ values above 1.0, demonstrating excellent broadband CPL detection ability suitable for potential applications in biosensing, molecular detection, and other fields^42,43^.

**Fig. 3:**
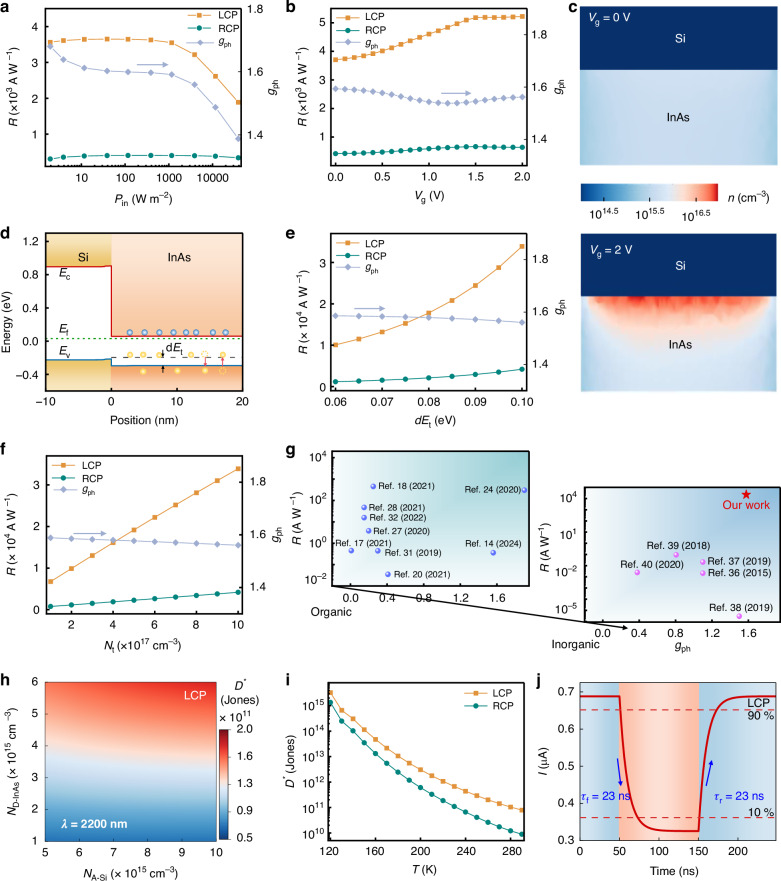
Enhancement of electrical gain and performance summary. **a** The *R* and *g*_ph_ of the device vs *P*_in_. **b** The *R* and *g*_ph_ of the device as a function of gate voltage (*V*_g_). **c** Spatial electron concentration distributions in the InAs and Si layers under different *V*_g_ for LCP light incidence. **d** Energy band diagram of the InAs/Si heterojunction. The *R* and *g*_ph_ of the devices vs the **e** trap energy-level depth (d*E*_t_) and **f** trap concentration (*N*_t_). **g** Summary of *R* and *g*_ph_ of the representative CPL detectors, where our work is also marked. **h** Relationship between the specific detectivity (D^*^) of the device and *n*-type InAs and *p*-type Si doping concentrations (*N*_D-InAs_ and *N*_A-Si_) at room temperature. **i** The D^*^ of the device as a function of operating temperature (*T*). **j** Response time of the device under LCP light incidence. Note that the default *λ*, *P*_in_, and *V*_ds_ are 2200 nm, 388.2 W cm^−2^, and 10 mV, respectively

Additionally, Fig. 2e reveals an intriguing observation wherein the *g*_ph_ surpasses *g*_abs_ (< 1.24) displayed in Fig. 1, signifying stronger electrical asymmetry compared to optical asymmetry. This phenomenon cannot be fully elucidated by the photoconductive effect alone, thus necessitating further investigation. Upon careful analysis, we attribute this anomaly to the photogating effect induced by the InAs/Si heterojunction. To validate this hypothesis, we explore the relationship between *R* and *P*_in_, as illustrated in Fig. 3a. It indicates that *R* initially increases slightly and then decreases with increasing *P*_in_, representing a typical phenomenon arising from the photogating effect^44^. This behavior occurs because increasing *P*_in_ reduces the depletion level in the InAs channel, thereby enhancing its conductivity and generating electrical gain, which subsequently enhances *R*. However, as *P*_in_ continues to increase, channel recombination intensifies due to the massive accumulation of electrons and holes, as depicted in Fig. S4, leading to a decline in *R*. Since *A*_L_InAs_ is significantly larger than *A*_R_InAs_, the *R* under LCP illumination decays more rapidly at higher *P*_in_, resulting in a decrease in *g*_ph_ as the *P*_in_ increases, as shown in Fig. 3a. Consequently, optimizing *R* and *g*_ph_ through the photogating effect requires operating the device under low *P*_in_. For instance, at a low *P*_in_ of 38.82 W cm^−2^, the device achieves peak values of *R* = 3600 A W^−1^ and *g*_ph_ = 1.68, as illustrated in Fig. 3a.

Analogous to the photogating effect, gate modulation provides an effective means to regulate device performance by incorporating an insulating gate atop the device. Figure 3b illustrates the relationship between the device’s *R* and *g*_ph_ vs the insulating gate bias (*V*_g_), demonstrating a significant increase in *R* with increasing *V*_g_, reaching about 5200 A W^−1^ at *V*_g_ = 2 V. This enhancement is attributed to the enhanced positive *V*_g_, which increases the vertical electric field, facilitating the separation of electron-hole pairs and reducing recombination, as shown in Fig. S5. Consequently, the non-equilibrium carrier concentration in the InAs channel rises, as depicted in Fig. 3c, leading to an improved *R*. Furthermore, within the *V*_g_ range of 0–2 V, *g*_ph_ exhibits slight fluctuations around 1.56, maintaining a consistently high value.

Furthermore, electrical gain and *R* can be further enhanced by introducing the trap effect. The trap effect stems from impurities or defects incorporated during material growth or device fabrication, such as lattice mismatches, dislocations, surface impurity adsorption, and interface defect states. Alternatively, it can be intentionally manipulated by introducing vacancies or impurities via molecular modifications, enabling control over trap concentrations and energy levels. According to trap effect theory, trap centers capture non-equilibrium minority carriers, prolonging their relaxation time and the effective lifetime of majority carriers, thereby enhancing electrical gain and *R*. Therefore, we introduce hole traps into the *n*-type InAs layer, with the trap energy level depicted by the black dashed line in Fig. 3d, where d*E*_t_ is the energy difference between the trap energy level and the valence band. Figures 3e, f illustrate the correlation between the device’s *R* and *g*_ph_ with respect to trap levels, encompassing both trap concentration (*N*_t_) and d*E*_t_. These findings reveal that as the d*E*_t_ and *N*_t_ increase, the *R* further increases, reaching up to 33,900 A W^−1^, while the *g*_ph_ experiences only a slight decrement, maintaining an elevated level above 1.56. This observation is attributed to the fact that higher *N*_t_ or deeper trap energy levels increase gain, leading to an increased *R*, as elaborated in the following trap effect theory^45,46^:

3 $$H=\frac{{N}_{{\rm{t}}}}{{N}_{{\rm{v}}}\exp \left(-\frac{{\rm{d}}{E}_{{\rm{t}}}}{{k}_{{\rm{B}}}T}\right)+\Delta p}$$

4 $${\tau }_{{\rm{eff}}}=\tau \left(1+H\right)$$

5 $${G}_{{\rm{a}}-\mathrm{trap}}={\tau }_{\mathrm{eff}}\left(\frac{1}{{\tau }_{\mathrm{tn}}}+\frac{1}{{\tau }_{\mathrm{tp}}}\right)$$

where *H* represents the trap accommodation capability for non-equilibrium minority carriers, which becomes significant when *H* is much greater than 1. *N*_V_, *K*_B_, *T*, ∆*p*, *τ*_eff_, and *G*_a-trap_ are the effective density of states in the valence band, Boltzmann constant, operating temperature (293 K), non-equilibrium minority carrier concentration, effective carrier lifetime induced by the trap effect, and electrical gain in the presence of the trap effect, respectively.

In a word, through the strategy of coupling inorganic chiral metasurfaces with electrical gain, our proposed CPL detector achieves both ultrahigh *R* and significant *g*_ph_ simultaneously. To situate the performance of our device within the broader context of the field, we conduct a comprehensive review of the performance metrics of representative CPL detectors reported in the literature, as summarized in Fig. 3g, with the detailed data provided in Table S2 of the Supporting Information. This comparative analysis underscores the superior overall performance of our proposed device in comparison to chiral organic materials-based CPL detectors. Moreover, compared to similar inorganic stable devices, our device exhibits substantial advantages in terms of both *R* and *g*_ph_.

In addition, the specific detectivity (D^*^), an important metric for evaluating photodetector sensitivity and noise characteristics, is expressed as follows:

6 $${D}^{* }=\frac{\sqrt{S* \Delta f}}{{NEP}}$$

7 $${NEP}=\frac{{i}_{{\rm{n}}}}{R}$$

8 $$< {i}_{{\rm{n}}}^{2} > =\left[2q < {I}_{\mathrm{dark}} > +\frac{4{K}_{B}T}{{R}_{\mathrm{shunt}}}+{i}_{1/{\rm{f}}}^{2}\right]\Delta f$$

where *Δf* is the electrical bandwidth, *i*_n_ is the total noise current, *NEP* is the noise equivalent power, <*I*_dark_> is the average dark current, *R*_shunt_ is the shunt resistance, and *i*_1/f_ is the 1/*f* noise current. The primary sources of noise include shot noise, thermal noise, and 1/*f* noise, with the latter being negligible at high frequencies. We further calculate the D^*^ spectra of the device at room temperature, as shown in Fig. S6. The results indicate that the device achieves a D^*^ on the order of magnitude of 10^10^ Jones within a broad spectral range, peaking at 7.47 × 10^10^ Jones at *λ* = 2200 nm. Further enhancement of D^*^ is achieved by adjusting the doping concentrations of *n*-type InAs and *p*-type Si (*N*_D-InAs_ and *N*_A-Si_). Figure 3h demonstrates that increasing doping concentrations, particularly *N*_D-InAs_, significantly improve D^*^. This improvement is attributed to an enhanced *R/I*_dark_ due to increasing photogating effect, as illustrated in Fig. S7. When *N*_D-InAs_ = 6 × 10^15 ^cm^−3^ and *N*_A-Si_ = 10^16 ^cm^−3^, the D^*^ reaches 1.8 × 10^11^ Jones, which represents excellent performance for mid-infrared detection at *λ* = 2200 nm under room-temperature conditions^47,48^. Additionally, as shown in Fig. 3i, decreasing the operating temperature (*T*) could further enhance D^*^ by several orders of magnitude. This is due to the suppression of thermal carrier excitation under low temperatures, resulting in a reduction in dark current, as depicted in Fig. S8. For a detailed analysis of how several factors influencing *I*_dark_ and *i*_n_ (such as *V*_ds_, doping concentration, and *T*) affect the *i*_n_, *NEP*, and signal-to-noise ratio (*SNR*), please see Fig. S9. Furthermore, the response time of the detector, defined as the time required for the signal current to rise from 10% (90%) to 90% (10%) or fall from 90% (10%) to 10% (90%), is another crucial performance parameter of photodetectors. Figure 3j displays the transient response current of the device. Under LCP light incidence, the rise time (*τ*_r_) and fall time (*τ*_f_) are both approximately 23 ns, which significantly outperforms previously reported CPL detectors, demonstrating the capability of the proposed devices for ultrafast detection.

### Self-powered mode

**Fig. 4:**
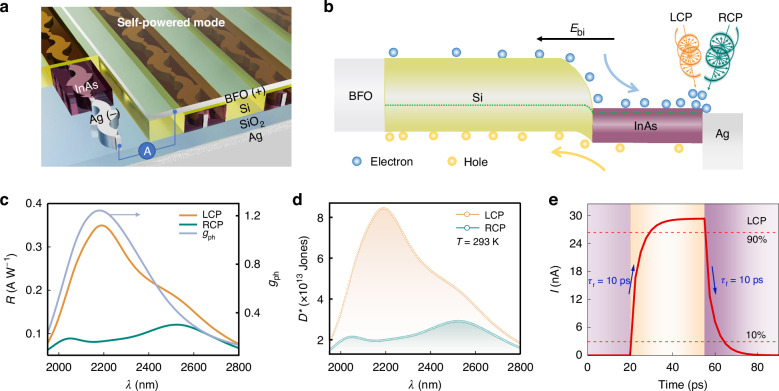
Self-powered CPL detection. **a** Schematic diagram of a self-powered photodiode CPL detector. **b** Energy band diagram of the photodiode CPL detector, where the *E*_bi_ is the built-in electric field. **c** The *R* and *g*_ph_ spectra of the device. **d** Specific detectivity (D^*^) of the device. **e** Response time of the device under LCP light incidence

Considering the advantages of energy savings, maintenance-free operation, and independence, certain application scenarios necessitate a self-powered mode. To address this need, we further explore the potential of our device for self-powered CPL detection. To achieve this, we introduce specific modifications to the device configuration. Specifically, the insulating Si_3_N_4_ is replaced with BFO, a transparent electrode material with a similar refractive index. The BFO layer and the chiral Ag nano-array serve as the top and bottom electrodes, respectively, while Si and InAs form a heterojunction. The device adopts a photodiode-type structure, facilitating carrier transport from top to bottom, as illustrated in Fig. 4a. The mechanism underlying the differential light absorption in this device aligns with that observed in the aforementioned photoconductive device. However, the electrical response mechanism differs significantly, being primarily driven by the built-in electric field (*E*_bi_) formed by band bending within the Si/InAs heterojunction, as depicted in Fig. 4b.

The electrical performance of the device is characterized and presented in Fig. 4c–e. Specifically, Fig. 4c shows the *R* and *g*_ph_ spectra. Under LCP light incidence, the device achieves a maximum *R* of 0.35 A W^−1^, which is 1–2 orders of magnitude higher than that of existing CPL detectors based on inorganic chiral structures with a hot electron injection mechanism^36–38^, meeting the requirements for commercial applications. Additionally, in the wavelength range of 1950–2800 nm, the *g*_ph_ initially increases and subsequently decreases, mostly exceeding 0.4 and reaching a maximum of 1.24 near 2200 nm. These results indicate that the self-powered CPL detector can effectively distinguish between LCP and RCP light. Subsequently, the D^*^ of the self-powered CPL detector is shown in Fig. 4d. The maximum D^*^ is approximately 8.5 × 10^13^ Jones, representing a relatively high level compared to other CPL detectors. Such a high D^*^ originates from the low dark current of the self-powered detector under zero-bias conditions. Figure 4e displays that under LCP light incidence, the rise time and fall time are both approximately 10 ps, with consistent response times observed under RCP light incidence, as detailed in Fig. S10. This ultrashort response time stems from the strong built-in electric field and the short carrier transport distance within the device. Consequently, the device demonstrates excellent performance in self-powered CPL detection, combining high *R*, superior D^*^, and ultrafast response time, thereby meeting the demands of high-performance applications.

### Encrypted communication

**Fig. 5:**
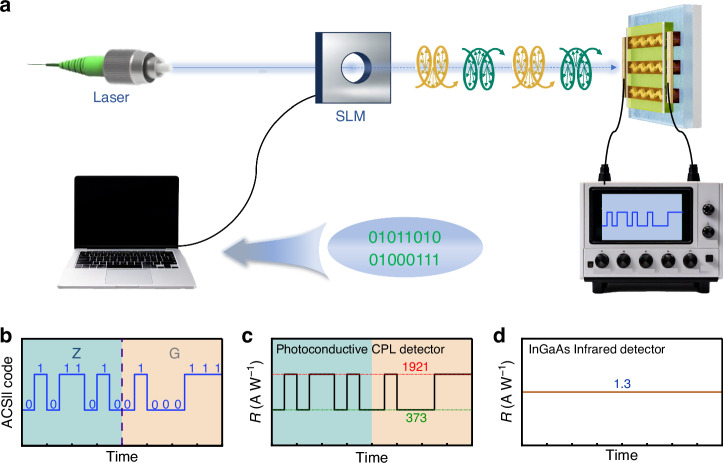
Encrypted communication. **a** Schematic diagram of the encrypted communication process. **b** ASCII code for the signal “ZG”. Response current waveforms from **c** the photoconductive CPL detector and **d** a commercial InGaAs infrared detector [https://www.hamamatsu.com/]

Based on the high-performance photoconductivity-type CPL detector, we demonstrate its specific application in encrypted communication, as illustrated in Fig. 5a. Taking the signal “ZG” as an example, it is first encoded into the ASCII code “01011010 01000111”, as shown in Fig. 5b. At the transmitting end, polarized light emitted by a laser with *λ* = 2200 nm serves as the signal carrier. The ASCII code of the signal is loaded onto the laser beam through a spatial light modulator (SLM), with a binary digit of 1 (0) corresponding to LCP (RCP) light. At the receiving end, the proposed CPL detector acts as the optical signal receiver and converter, generating polarization-dependent current signals, as depicted in Fig. 5c. Specifically, the detector produces a *R* of 1921 A W^−1^ under LCP light and 373 A W^−1^ under RCP light. Utilizing the current amplitudes and the ASCII code table, the intended recipient can directly and securely obtain the information without requiring an encryption key^49^. Conversely, an adversary attempting to intercept the communication using commercially available infrared detectors would only capture a non-fluctuating electrical signal, as shown in Fig. 5d, making it nearly impossible to decipher the valid information. This proposed key-free and straightforward encryption scheme offers convenience and enhanced security for optical encryption communication systems. In practical applications, there may be challenges such as interference and depolarization. To deal with these issues, a multidimensional technology integration approach is essential. Notably, artificial intelligence may also provide breakthrough solutions.

## Discussion

This work proposed a CPL detector based on chiral Ag nanowires/InAs/Si, which enables simultaneous achievement of ultra-high *R* and *g*_ph_ while maintaining device stability by coupling a chiral metasurface with electrical gain. Optically, the differential interaction of “S”-shaped chiral Ag nanowires with LCP and RCP light enables the device to obtain a *g*_abs_ as high as 1.24 at *λ* = 2200 nm. It should be noted that, theoretically, it is possible to further increase the *g*_abs_. To reach or approach this point, advanced optimization algorithms can be combined with precise numerical simulations in the future to approximate the maximum limit of *g*_abs_. Electrically, the device benefits from substantial electrical gain arising from the photoconductive effect assisted by mechanisms such as photogating, gate modulation, and trap effects, achieving an ultrahigh *R* of 33,900 A W^−1^ and *g*_ph_ of above 1.56 under a bias of 10 mV, far exceeding previously reported performances. Additionally, the device can also operate in a self-powered mode, achieving a D^*^ of 10^13^ Jones and an ultra-short response time of 10 ps, enhancing its potential for low-power and convenient applications. Finally, the application of this device in secure communication systems was demonstrated, where its capability to distinguish between LCP and RCP light enables secure, keyless optical encryption. This property makes the detector highly suitable for encrypted communication, especially in fields with high security requirements such as military communications and financial transactions. In summary, by combining inorganic chiral metasurfaces with electrical gain, the proposed CPL detector not only advances the state-of-the-art in polarized optical detection but also paves the way for applications in next-generation secure communication systems.

## Materials and methods

In this study, a rigorous three-dimensional optoelectronic coupling simulation model based on the finite element method in frequency or time domains was constructed to accurately simulate the optoelectronic response performance of the device. In this simulation, the interaction between light and matter was calculated by rigorously solving Maxwell’s equations, to obtain the electromagnetic field loss distribution within the device, as well as the absorptivity, transmissivity, reflectivity, and the spatial distribution of photogenerated carriers. Furthermore, the generation, recombination, transport, and collection processes of the carriers were accurately simulated by rigorously solving the semiconductor continuity equations and Poisson’s equation. This full-space multiphysics simulation enabled the accurate determination of both the electrical and optoelectronic responses of the device. The core equations are shown as follows:

9 $$\nabla \cdot \left(-{D}_{{\rm{n}}}\nabla n+n{\mu }_{{\rm{n}}}\nabla \Phi \right)=G-U$$

10 $$\nabla \cdot \left(-{D}_{{\rm{p}}}\nabla p-p{\mu }_{{\rm{p}}}\nabla \Phi \right)=G-U$$

11 $${\nabla }^{2}\Phi =\,\frac{q}{{\varepsilon }_{0}{\varepsilon }_{{\rm{r}}}}\left(n-p+{N}_{{\rm{a}}}-{N}_{{\rm{d}}}\right)$$

12 $$U={U}_{{\rm{rad}}}+{U}_{{\rm{aug}}}+{U}_{{\rm{SRH}}}$$

13 $${U}_{{\rm{rad}}}={B}_{{\rm{rad}}}({np}-{n}_{{\rm{i}}}^{2})$$

14 $${U}_{{\rm{aug}}}=({A}_{{\rm{n}}}n+{A}_{{\rm{p}}})({np}-{n}_{{\rm{i}}}^{2})$$

15 $${U}_{{\rm{SRH}}}=\frac{{np}-{n}_{{\rm{i}}}^{2}}{\left(n+{n}_{{\rm{lr}}}\right){\tau }_{{\rm{r}}}+\left(p+{p}_{{\rm{lr}}}\right){\tau }_{{\rm{r}}}}$$

where *D*_n_/*D*_p_ (cm^2^/s) is the diffusion coefficient of electron/hole, *n*/*p* (cm^−3^) is the electron/hole concentration, *μ*_n_/*μ*_p_ (cm^2^ V^−1^ s^−1^) is the mobility of electron/hole, *Φ* (V) is the electrostatic potential, *ε*_0_/*ε*_r_ is the vacuum/relative dielectric constant, *N*_d_/*N*_a_ (cm^−3^) is the concentration of the donor/acceptor. *U* (cm^−3^ s^−1^) is the total recombination rate, including *U*_rad_/*U*_aug_/*U*_SRH_, representing the radiative/Auger/Shockley-Read-Hall (SRH) recombination rate, respectively. *B*_rad_ stands for the radiative recombination coefficient, *A*_n_/*A*_p_ denotes the electron/hole Auger recombination coefficient, *n*_i_ is the intrinsic electron concentration, *n*_1r_/*p*_1r_ is the electron/hole concentration within the trap state (the strongest *U*_SRH_ occurs when *n*_1r_ = *p*_1r_ = *n*_i_), and *τ* is the carrier lifetime (refer to Table S1). It should be noted that in InAs, the SRH recombination dominates. Further details regarding the simulation methods can be found in our previous publications^46,49,50^. Although this study is based on theoretical simulations, we provide a feasible fabrication scheme for the “S”-shaped chiral Ag nanowires, as shown in section S14.

## Supplementary information

Supporting information

## Data Availability

The authors declare that the data supporting the findings of this study are available with the paper and its Supplementary Information files. The data that support the findings of this study are available from the corresponding author upon reasonable request.
