# Supplementary material for: Electrical-gain-assisted circularly polarized photodetection based on chiral plasmonic metamaterials
Source: Light Sci Appl. 2025 Aug 11;14:265. doi: 10.1038/s41377-025-01932-9 (PMC12339686; doi:10.1038/s41377-025-01932-9)
Supplement: Supplementary file 1 — Supporting information [file 41377_2025_1932_MOESM1_ESM.docx]

*Supporting information for*

**Electrical-gain-assisted circularly polarized photodetection based on chiral plasmonic metamaterials**

Chenghao Chen^1,2^, Zhenhai Yang^1,2,*^, Tianyi Hang^1,2^, Yining Hao^1,2^, Yijing Chen^1,2^ , Chengzhuang Zhang^1,2^, Jiong Yang^1,2^, Xiaoyi Liu^1,2^, Xiaofeng Li^1,2,*^, and Guoyang Cao^1,2,3,*^

^1^School of Optoelectronic Science and Engineering & Collaborative Innovation Center of Suzhou Nano Science and Technology, Soochow University, Suzhou 215006, China

^2^Key Laboratory of Advanced Optical Manufacturing Technologies of Jiangsu Province & Key Laboratory of Modern Optical Technologies of the Ministry of Education, Soochow University, Suzhou 215006, China

^3^Engineering Research Center of Digital Graphic and Next-Generation Printing, Jiangsu Province, Soochow University, Suzhou 215006, China

^*^Corresponding author: zhyang@suda.edu.cn; xfli@suda.edu.cn; gycao@suda.edu.cn

**Contents:**

**S1.** **Detailed optimization process of device structure**

**S2.** **Discussion about optical asymmetry mechanism**

**S3. Dimensional robustness**

**S4. Approximate voltage range for the linear operation region**

**S5.** **Recombination enhancement in InAs with increasing *P*_in_**

**S6.** **Vertical electric field density and recombination current in InAs**

**S7. *D*^*^ spectra of the devices at room temperature**

**S8. The** **influence of doping concentration of InAs and Si on device performance**

**S9.** **The** **influence of ambient temperature on *I*_dark_**

**S10.** **The influence of *V*_ds_, doping concentration, and *T* on the *i*_n_, *NEP*, and signal-to-noise ratio (*SNR*)**

**S11.** **Transient response of photodiode device under RCP incidence**

**S12. Semiconductor parameters of InAs and Si**

**S13. Performance summary of CPL detectors**

**S14. A feasible fabrication scheme for the “S”-shaped chiral Ag nanowires**

**S1. Detailed optimization process of device structure**

**Figure S1.** Schematic diagram of the CPL photodetector with (a) “X”-shaped chiral Ag structure, (b) “S”-shaped chiral without sharp corner and (c) “S”-shaped chiral with sharp corner Ag structure. The absorptivity of InAs in the device with (d) the “X”-shaped chiral Ag structure, (e) “S”-shaped chiral without sharp corner and (f) “S”-shaped chiral with sharp corner Ag structure. The absorptivity spectra of InAs in the device with “S”-shaped chiral Ag structure under different *θ*_2_ at (g) LCP and (h) RCP incidence. (i) The *g*_abs_ spectra of the device with “S”-shaped chiral with sharp corner Ag structure under different *θ*_2_. Here, *g*_abs_ is the optical asymmetry factor defined as 2(*A*_L_InAs_ – *A*_R_InAs_)/(*A*_L_InAs_ + *A*_R_InAs_), where *A*_L_InAs_ (*A*_R_InAs_) is the absorptivity of the InAs layer under LCP (RCP) light incidence.

**S2.** **Discussion about optical asymmetry mechanism**


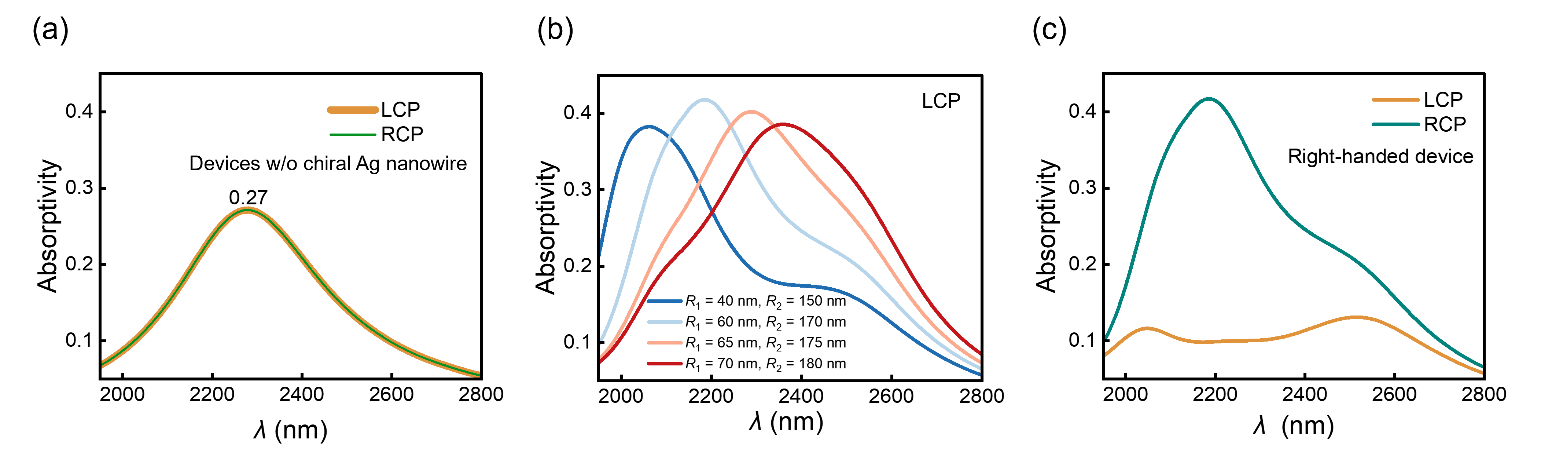


**Figure S2.** (a) The absorptivity of the InAs in the device without chiral Ag nanowire under LCP and RCP incidence. (d) Resonance peak shifts in the absorption spectra. (c) The absorptivity of InAs in right-handed device under LCP and RCP incidence.

**S3.** **Dimensional robustness of the device**

**Figure S3.** (a) *g*_abs_ as a function of thickness of Si (*H*_Si_). (b) *g*_abs_ as a function of thickness and width of InAs (*H*_InAs_ and *W*_InAs_).

**S4. Approximate voltage range for the linear operation region.**

The *v*_s_ can be calculated using Equation S1. Through Equation S2, we further determine the electric field intensity range corresponding to the linear region. By employing the approximation of a uniformly distributed electric field in Equation S3, we derive an approximate voltage range of 0-88.6 mV for the linear operation region.

$\frac{3}{2}K_{B}T=\frac{1}{2}mv_{s}^{2}$ (S1)

$v_{s}=\mu E_{\max}$ (S2)

$V_{\max}=W_{2}\times E_{\max}$ (S3)

where *K*_B_ is the Boltzmann constant, *T* is the temperature unit Kelvin, and *m* is the effective mass of carriers. The *μ* is the carrier mobility and *E*_max_ is the largest electric field strength for the linear region. *W*_2_ is the distance between the two electrodes and *V*_max_ is the largest voltage of the linear region.

**S5.** **Recombination enhancement in InAs with increasing *P*_in_**

**Figure S4.** The ratio of recombination rate to photo-excited carriers generation rate (*U*/*G*) as a function of *P*_in_.

**S6.** **Vertical electric field density and** **recombination current in InAs**

**Figure S5.** (a) Electric field vector (red arrow) distribution in InAs and Si. (b) Vertical electric field density with different *V*_g_, where position of 0 nm represents the interface between Si and InAs. (c) Recombination current in InAs as a function of *V*_g_.

**S7.** ***D*^*^ spectra of the devices at room temperature**

**Figure S6.** Specific detectivity (*D*^*^) spectra of the devices at room temperature.

**S8.** **The** **influence of doping concentration of InAs and Si on performance of the device**

**Figure S7.** The ratio of responsivity (*R*) to dark current (*I*_dark_) in relation to *n*-type InAs and *p*-type Si doping concentrations (*N*_D-InAs_ and *N*_A-Si_).

**S9.** **The** **influence of operating temperature on dark current**

**Figure S8.** The influence of operating temperature (*T*) on dark current (*I*_dark_).

**S10.** **The influence of *V*_ds_, doping concentration, and *T* on the *i*_n_, *NEP*, and signal-to-noise ratio (*SNR*)**

Here, we display an analysis of how several factors affecting dark current and noise current (such as *V*_ds_, doping concentration, and operating temperature *T*) influence the noise current (*i*_n_), noise-equivalent power (*NEP*), and signal-to-noise ratio (*SNR*), as shown in Figure S9. The corresponding formula for *SNR* is provided in Equations S4. Specifically, Figure S9a demonstrates that *i*_n_ increases monotonically with increasing *V*_ds_, primarily attributed to the enhancement of *I*_dark_. Figure S9b reveals that *NEP* decreases with increasing *V*_ds_, indicating a negative correlation with *i*_n_. This is attributed to the *V*_ds_-induced electrical gain, which enhances the responsivity (*R*), as shown in Equation 7. Figure S9c shows that *SNR* increases with increasing *V*_ds_, as the electrical gain induced by *V*_ds_ boosts the response current. Notably, under LCP illumination, both the response current and *R* are higher compared to RCP illumination. Consequently, within the tested *V*_ds_ range, *NEP* under LCP illumination remains consistently lower, while *SNR* remains higher. Figures S9d, e, and f depict the effects of doping concentrations in *n*-type InAs and *p*-type Si (*N*_D-InAs_ and *N*_A-Si_) on *i*_n_, *NEP*, and *SNR*. The results indicate that increasing *N*_A-Si_ while reducing *N*_D-InAs_ effectively decreases *i*_n_, as a wider InAs channel depletion region and a lower thermally stable carrier concentration in the InAs channel are beneficial in this regard. Among these factors, *N*_D-InAs_​ has the most significant impact on *NEP* and *SNR*: a higher *N*_D-InAs_ leads to a lower *NEP* and a higher *SNR* due to the enhanced photogating effect, which increases the ratio of *R/I*_dark_, as shown in Figure S7. Figures S9g, h, and i demonstrate that *i*_n_ and *NEP* increase with temperature, whereas *SNR* decreases. This highlights the advantage of lower temperatures in optimizing device performance.

$SNR (dB)=20\log_{10}(\frac{I_{\mathrm{ph}}}{i_{n}})$ (S4)

**Figure S9.** (a) The *i*_n_ as a function of *V*_ds_. The (b) *NEP* and (c) *SNR* as a function of *V*_ds_ under LCP and RCP incidence with a light power density (*P*_in_) of 38820 W m^−2^. (d) The *i*_n_ of the device as a function of doping concentrations of *n*-type InAs and *p*-type Si (*N*_D_-InAs and *N*_A_-Si) at room temperature. The (e) *NEP* and (f) *SNR* as a function of *N*_D_-InAs and *N*_A_-Si at room temperature under LCP incidence with a *P*_in_ of 388.2 W m^−2^. (g) The *i*_n_ as a function of operating temperature (*T*). The (h) *NEP* and (i) *SNR* as a function of *T* under LCP and RCP incidence with a *P*_in_ of 388.2 W m^−2^.

**S11.** **Transient response of photodiode device under RCP incidence**

**Figure S10.** Transient response of left-handed devices under RCP incidence.

**S12. Semiconductor parameters of InAs and Si**

**Table S1.** Semiconductor parameters of InAs and Si.

| Materials | *E*_g_ (eV) | *χ* (eV) | *ε*_r_ | *N*_C_/*N*_V_ (cm^−3^) | *μ*_n_/*μ*_p_ (cm^2^ V^−1^ s^−1^) | *τ* (ns) |
| --- | --- | --- | --- | --- | --- | --- |
| Si | 1.12 | 4.05 | 11.7 | 2.7×10^19^/1.0×10^19^ | 1450/500 | 10^4^ |
| InAs | 0.354 | 4.9 | 15.15 | 8.4×10^16^/6.4×10^18^ | 40000/500 | 10 |

**S13. Performance summary of CPL detectors**

| **Type** | **Literature** | ***g*_ph_** | ***R* (A W^−1^)** | ***D*^*^ (Jones)** | ***τ*_r_/*τ*_f_ (ms)** | ***λ*_max_**  **(nm)** |
| --- | --- | --- | --- | --- | --- | --- |
| Inorganic devices | **Our work** | **1.56** | **3.39×10^4^** | **1.8×10^11^** | **2.3×10^−5^/**  **2.3×10^−5^** | **2200** |
|  | [1] *Nat Commun.* 6, 8379 (2015) | 1.1 | 2.1×10^−3^ | / | / | 1350 |
|  | [2] *Phys. Scr*. 94, 085501 (2019) | 1.2 | 2.1×10^−2^ | / | / | 1550 |
|  | [3] *Opt. Lett.* 44, 2998–3001 (2019) | 1.5 | 3.0×10^−7^ | / | / | 4250 |
|  | [4] *Nanoscale* 12, 5906−5913 (2020) | 0.06 | 2.46×10^−3^ | / | / | 790 |
| Organic devices | [5] *Nat. Photon.* 7, 634–638 (2013) | 1.6 | 1×10^−4^ | / | 2.6/6.2 | 365 |
|  | [6] *Nat. Commun.* 12, 142 (2021) | 0.01 | 0.45 | 2.1×10^10^ | / | 730 |
|  | [7] *Nat. Commun.* 10, 1927 (2019) | 0.1 | 0.12 | 7.1×10^11^ | / | 395 |
|  | [8] *Nano Today* 54, 102132 (2024) | 1.56 | 0.501 | 7.12×10^12^ | 0.65/5.7 | 870 |
|  | [9] *Adv. Sci.* 10, 2206070 (2023) | / | 8.1×10^−5^ | 1.2×10^9^ | 9.0×10^−2^/  9.0×10^−2^ | 800 |
|  | [10] *Adv. Funct. Mater.* 34, 2311726 (2024) | 0.56 | / | / | / | 405 |
|  | [11] *ACS Nano* 16, 2682−2689 (2022) | 0.15 | 16 | / | / | 405 |
|  | [12] *ACS Nano* 15, 7608−7617 (2021) | 0.21 | 4.5×10^2^ | / | / | 405 |
|  | [13] *Angew. Chem. Int. Ed.* 60, 8415 (2021) | 0.3 | 2.2×10^−5^ | 1.2×10^7^ | 580/960 | 520 |
|  | [14] *ACS Nano* 14, 14146−14156 (2020) | 0.13 | 1.2×10^3^ | 2.9×10^13^ | <50/<50 | 495 |
|  | [15] *J. Mater. Chem. C* 8, 9271–9275 (2020) | 1.9 | 4.6×10^−6^ | 1.5×10^7^ | / | 450 |
|  | [16] *Adv. Funct. Mater*. 30, 2006236 (2020) | 1.9 | 3.0×10^2^ | / | 260/310 | 830 |
|  | [17] *ACS Nano* 13, 3659−3665 (2019) | 0.3 | 0.44 | 2.2×10^11^ | 100/100 | 518 |
|  | [18] *ACS Nano* 13, 9473−9481 (2019) | 0.2 | 0.6 | 3.1×10^11^ | 22/34 | 520 |
|  | [19] *Adv. Funct. Mater.* 29, 1808668 (2019) | 0.1 | 5.4×10^−4^ | / | / | 375 |
|  | [20] *Adv. Funct. Mater.* 29, 1900684 (2019) | 0.09 | 0.21 | / | / | 543 |

**Table S2.** A summary of high-performance CPL detectors.

**S14. A feasible fabrication scheme for the “S”-shaped chiral Ag nanowires**

The possible fabrication process of the “S”-shaped chiral Ag nanowires is outlined as follows: a) Sequential deposition of Ag, SiO_2_ and Si layers onto the Si wafers using electron beam evaporation, with the thicknesses of ~300 nm, 150 nm, and 300 nm, respectively. b) Spin-coating a thin (~50 nm) hydrogen silsesquioxane (HSQ) negative photoresist layer onto the top Si layer. c) Defining complementary nanopatterns of the “S”-shaped chiral Ag nanowires onto the HSQ photoresist using EBL. The unexposed areas are removed after development. d) Performing inductively coupled plasma (ICP) etching on the Si layer beneath the photoresist, stopping at the underlying SiO_2_ layer. e) Depositing ~120 nm of Ag into the etched “S”-shaped trench *via* electron beam evaporation. f) Removing the sacrificial Si layers through wet etching with KOH or TMAH. By carefully controlling the etching conditions, the “S”-shaped chiral Ag nanowires are successfully formed on the SiO_2_/Ag substrate.


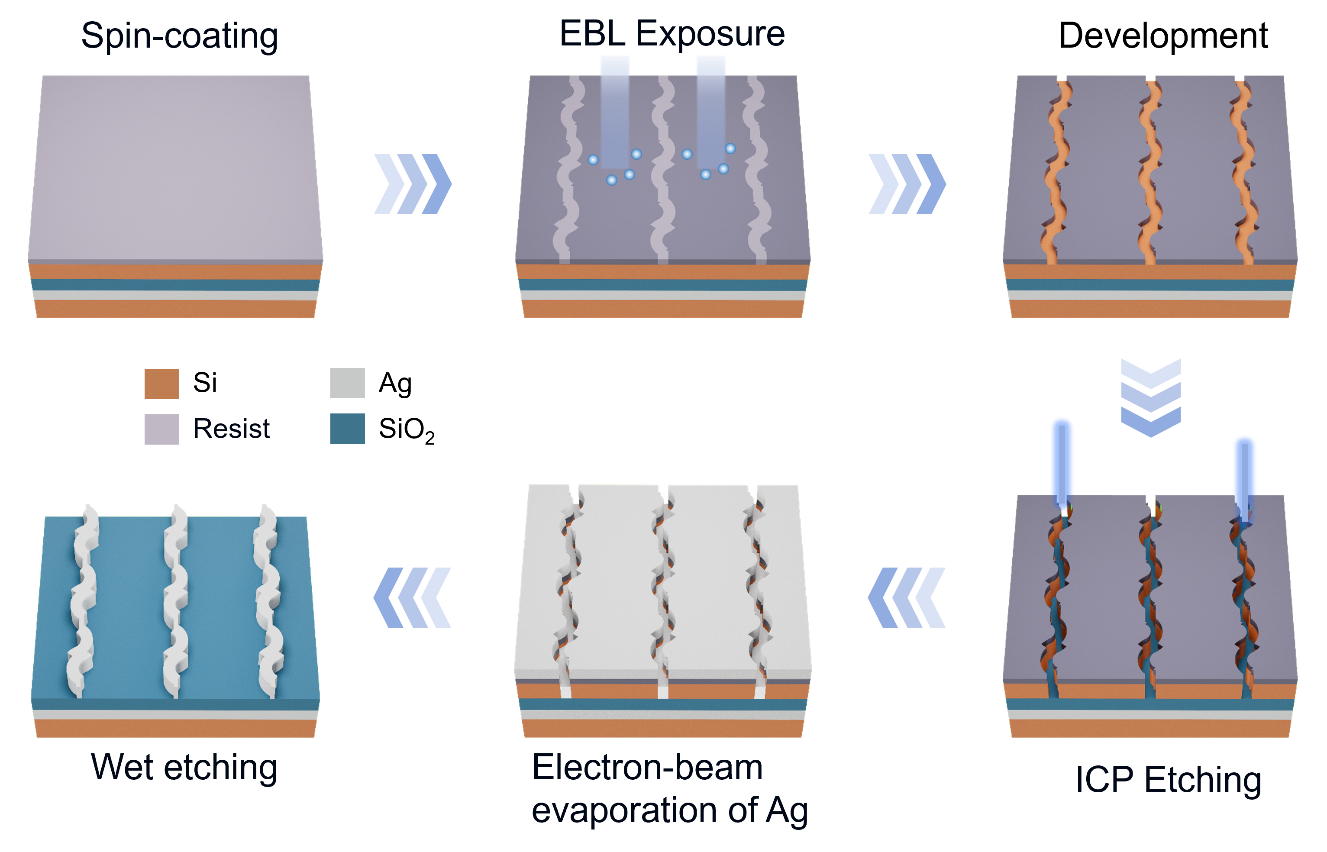


Figure S11. Flow chart of feasible preparation scheme of “S”-shaped chiral Ag nanowires.

**References**

1. Li, W. et al. Circularly polarized light detection with hot electrons in chiral plasmonic metamaterials. *Nat Commun.* **6**, 8379 (2015).
2. Xiao, W. et al. Circularly polarized light detector based on 2D embedded chiral nanostructures. *Phys. Scr*. **94**, 085501 (2019).
3. Peng, J. et al. Direct detection of photon spin angular momentum by a chiral graphene mid-infrared photodetector. *Opt. Lett.* **44**, 2998–3001 (2019).
4. Jiang, Q. et al. Ultrathin circular polarimeter based on chiral plasmonic metasurface and monolayer MoSe_2_. *Nanoscale* **12**, 5906−5913 (2020).
5. Yang, Y. et al. Circularly polarized light detection by a chiral organic semiconductor transistor. *Nat. Photon.* **7**, 634–638 (2013).
6. Zhang, L. et al. π-Extended perylene diimide double-heterohelicenes as ambipolar organic semiconductors for broadband circularly polarized light detection. *Nat. Commun.* **12**, 142 (2021).
7. Chen, C. et al. Circularly polarized light detection using chiral hybrid perovskite. *Nat. Commun.* **10**, 1927 (2019).
8. Wang, Q. et al. High-performance near-infrared narrowband circularly polarized light organic photodetectors. *Nano Today* **54**, 102132 (2024).
9. Wu, W. et al. Toward efficient two-photon circularly polarized light detection through cooperative strategies in chiral quasi-2D perovskites. *Adv. Sci.* **10**, 2206070 (2023).
10. Chen, Y. et al. Inch-size achiral perovskite single crystals for distinguishing circularly polarized light with a large asymmetry factor. *Adv. Funct. Mater.* **34**, 2311726 (2024).
11. Liu, T. et al. High responsivity circular polarized light detectors based on quasi two-dimensional chiral perovskite films. *ACS Nano* **16**, 2682−2689 (2022).
12. Hao, J. et al. Direct detection of circularly polarized light using chiral copper chloride-carbon nanotube heterostructures. *ACS Nano* **15**, 7608−7617 (2021).
13. Li, D. et al. Chiral lead-free hybrid perovskites for self-powered circularly polarized light detection. *Angew. Chem. Int. Ed.* **60**, 8415 (2021).
14. Shang, X. et al. Surface-Doped Quasi-2D Chiral Organic Single Crystals for Chiroptical Sensing. *ACS Nano* **14**, 14146−14156 (2020).
15. Cheng, J. et al. Enabling discrimination capability in an achiral F6BT-based organic semiconductor transistor via circularly polarized light induction. *J. Mater. Chem. C* **8**, 9271–9275 (2020).
16. Han, H. et al. High-performance circularly polarized light-sensing near-infrared organic phototransistors for optoelectronic cryptographic primitives. *Adv. Funct. Mater*. **30**, 2006236 (2020).
17. Ma, J. et al. Chiral 2D perovskites with a high degree of circularly polarized photoluminescence. *ACS Nano* **13**, 3659−3665 (2019).
18. Wang, J. et al. Aqueous synthesis of low-dimensional lead halide perovskites for room-temperature circularly polarized light emission and detection. *ACS Nano* **13**, 9473−9481 (2019).
19. Kim, N. Y. et al. Chiroptical-conjugated polymer/chiral small molecule hybrid thin films for circularly polarized light-detecting heterojunction devices. *Adv. Funct. Mater.* **29**, 1808668 (2019).
20. Schulz, M. et al. Chiral excitonic organic photodiodes for direct detection of circular polarized light. *Adv. Funct. Mater.* **29**, 1900684 (2019).
